# Complete chloroplast genome sequence of a tree fern *Alsophila spinulosa*: insights into evolutionary changes in fern chloroplast genomes

**DOI:** 10.1186/1471-2148-9-130

**Published:** 2009-06-11

**Authors:** Lei Gao, Xuan Yi, Yong-Xia Yang, Ying-Juan Su, Ting Wang

**Affiliations:** 1 Wuhan Botanical Garden, Chinese Academy of Sciences, Wuhan, Hubei 430074, PR China; 2 Graduate School, Chinese Academy of Sciences, Beijing 100039, PR China; 3 State Key Laboratory of Biocontrol, School of Life Sciences, Sun Yat-sen University, Guangzhou, 510275, PR China

## Abstract

**Background:**

Ferns have generally been neglected in studies of chloroplast genomics. Before this study, only one polypod and two basal ferns had their complete chloroplast (cp) genome reported. Tree ferns represent an ancient fern lineage that first occurred in the Late Triassic. In recent phylogenetic analyses, tree ferns were shown to be the sister group of polypods, the most diverse group of living ferns. Availability of cp genome sequence from a tree fern will facilitate interpretation of the evolutionary changes of fern cp genomes. Here we have sequenced the complete cp genome of a scaly tree fern *Alsophila spinulosa *(Cyatheaceae).

**Results:**

The *Alsophila *cp genome is 156,661 base pairs (bp) in size, and has a typical quadripartite structure with the large (LSC, 86,308 bp) and small single copy (SSC, 21,623 bp) regions separated by two copies of an inverted repeat (IRs, 24,365 bp each). This genome contains 117 different genes encoding 85 proteins, 4 rRNAs and 28 tRNAs. Pseudogenes of *ycf66 *and *trnT-UGU *are also detected in this genome. A unique *trnR-UCG *gene (derived from *trnR-CCG*) is found between *rbcL *and *accD*. The *Alsophila *cp genome shares some unusual characteristics with the previously sequenced cp genome of the polypod fern *Adiantum capillus-veneris*, including the absence of 5 tRNA genes that exist in most other cp genomes. The genome shows a high degree of synteny with that of *Adiantum*, but differs considerably from two basal ferns (*Angiopteris evecta *and *Psilotum nudum*). At one endpoint of an ancient inversion we detected a highly repeated 565-bp-region that is absent from the *Adiantum *cp genome. An additional minor inversion of the *trnD-GUC*, which is possibly shared by all ferns, was identified by comparison between the fern and other land plant cp genomes.

**Conclusion:**

By comparing four fern cp genome sequences it was confirmed that two major rearrangements distinguish higher leptosporangiate ferns from basal fern lineages. The *Alsophila *cp genome is very similar to that of the polypod fern *Adiantum *in terms of gene content, gene order and GC content. However, there exist some striking differences between them: the *trnR-UCG *gene represents a putative molecular apomorphy of tree ferns; and the repeats observed at one inversion endpoint may be a vestige of some unknown rearrangement(s). This work provided fresh insights into the fern cp genome evolution as well as useful data for future phylogenetic studies.

## Background

The chloroplast (cp) genome has long been a focus of research in plant molecular evolution and systematics due to its small size, high copy number, conservation and extensive characterization at the molecular level [1]. More recently, with technical advances in DNA sequencing, the number of completely sequenced cp genomes has grown rapidly. Aside from providing information on genome structure, gene content, gene order and nucleotide composition, complete cp genome sequences also offer a unique opportunity to explore the evolutionary changes of the genome itself. In general, cp genomes are structurally highly conserved across land plants. However, structural rearrangements, e.g. gene loss, inverted repeat (IR) loss or expansion and inversion, do occur in certain lineages and have been shown to be extremely informative in resolving deep phylogenetic relationships because they may exhibit less homoplasy than sequence data [1]. For example, a 30-kb inversion shared by all vascular plants except lycopsids identifies the lycopsids as the basal lineage in the vascular plants [2]. Two inversions and an IR expansion can be used to clarify basal nodes in the leptosporangiate ferns [3,4].

Currently, one limiting factor in comparative chloroplast genomics is the sparse taxon sampling in spore-bearing land plants. The representation of genome sequencing almost always favors plants of economic interest [5]. Complete cp genomes have been sequenced for more than one hundred seed plants. Amongst these, more than 10 completed sequences each are from cereals (13), crucifers (12) and conifers (12) respectively (see Additional file 1). But for other land plants, excluding seed plants, only 10 cp genome sequences have been achieved in total, of which only 3 are from ferns prior this study (see Additional file 1). For further insights into the evolutionary dynamics of cp genome organization, more data from plant species representative of other crucial evolutionary nodes is needed [5].

Ferns (monilophytes), with more than 10,000 living species, are the most diverse group of seed-free vascular plants [6,7]. Previous studies have uncovered considerable genomic rearrangements in fern cp genomes, but the details and exact series of these events have not yet been fully characterized [3,4,8]. The completed cp genome sequence of the polypod fern *Adiantum capillus-veneris *shows some unusual features not seen in vascular plants before, including tRNA gene losses, which had only been observed in cp genomes of non-photosynthetic plants [9,10]. For example, a putative *tRNA-selenocysteine *(*tRNA-Sec*) gene in *Adiantum *[10] replaces the typical *trnR-CCG *gene. Unfortunately, because *Adiantum *is the only sequenced representative of leptosporangiates, the most diverse fern lineage, it is difficult to tell which characteristics are unique to *Adiantum *or diagnostic of a much larger clade. Therefore, complete cp genome data from more fern clades are necessary to better resolve these issues.

As part of an effort to shed more light on the cp genome evolution in ferns, we have sequenced the complete plastid genome of a scaly tree fern *Alsophila spinulosa *(ab. *Alsophila*) (Cyatheaceae). This taxon was chosen because it is an easily available representative of an ancient lineage – tree ferns, for which no cp genome has been sequenced before. In addition to tree ferns, heterosporous and polypod ferns are the other two main lineages within the "core leptosporangiates" [11]. The three major lineages of "core leptosporangiates" were thought to have originated from a Late Triassic diversification [11]. Recent phylogenetic studies further demonstrated a sister relationship between tree ferns and polypods [6,11,12]. After the Late Triassic diversification, polypods remarkably re-diversified along with angiosperms in the Cretaceous [6,11]. Similarly, the scaly tree ferns (Cyatheaceae) also radiated very recently and diversified at an exceptionally high rate [13]. A comparison of the complete cp genome sequences between *Alsophila *and the polypod fern *Adiantum *will aid interpretation of unusual characters observed in *Adiantum*, such as some missing and novel genes [9,10].

Moreover, sequences of all four published fern cp genomes (including that of *Alsophila*) will enable more detailed comparisons of the organization and evolution of the chloroplast genomes in ferns. Our comparative analyses corroborate that fern cp genomes have undergone substantial changes in gene orders during evolution: two main rearrangements contribute to major differences between "higher" and basal ferns. In addition, the comparisons also identify some unique characteristics in the *Alsophila *cp genome including a novel tRNA, interesting pseudogenes and a highly repeated 565-bp-region spanning one endpoint of an ancient inversion.

## Results and Discussion

### General Features

**Figure 1:**
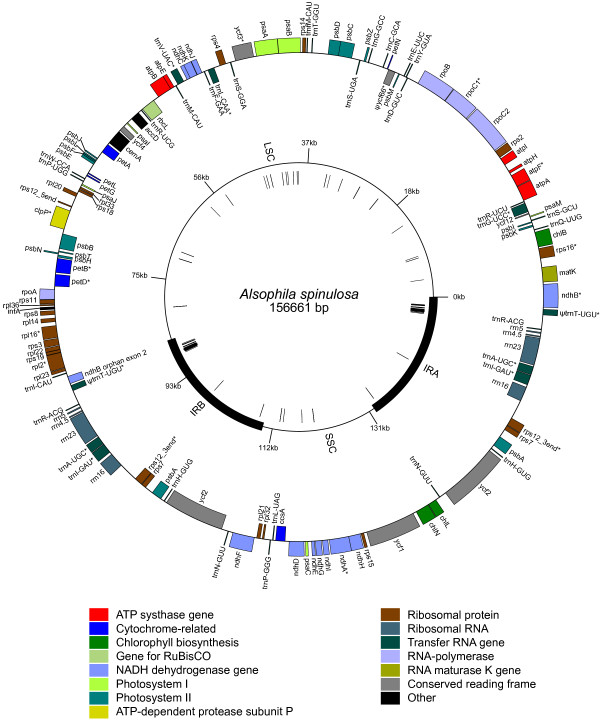
**Gene map of the *Alsophila spinulosa *chloroplast genome**. Thick black lines on inner cycle indicate the inverted repeats (IRA and IRB) which separate the genome into the large (LSC) and small (SSC) single copy regions. Genes shown on the inside of the circle are transcribed counterclockwise and those on the outside clockwise. Gene boxes are color coded by functional group as shown in the key. Asterisks denote genes with introns. Ψ represents pseudogene. Nucleotide positions are numbered starting at the boundary of IRA and LSC, with position 1 in the intron of *ndhB*. The circle of hashmarks indicates the location of direct and inverted repeats detected by REPuter [28].

The chloroplast (cp) genome of *Alsophila spinulosa *[GenBank: FJ556581] is 156,661 base pairs (bp) with a large single copy (LSC) region of 86,308 bp separated from a 21,623-bp small single copy (SSC) region by two inverted repeats (IRs), each of 24,365 bp (Figure 1). The genome is the largest amongst the four sequenced fern cp genomes (Table 1), but is smaller than previous estimates of other Cyatheaceae species, e.g. *Alsophila bryophila *(165 kb), *Cyathea furfuracea *(179.2 kb) and *Sphaeropteris cooperi *(164.3 kb), using the mapping method [14]. When the IR is considered only once, the *Alsophila *cp genome contains 117 genes, encoding 85 proteins, 4 rRNAs and 28 tRNAs (Table 1). Pseudogenes of *ycf66 *and *trnT-UGU *were also detected in this genome (Figure 1). More than half of the *Alsophila *cp genome is composed of coding regions (92,691 bp, 59.17%) with the protein-coding regions accounting for the major portion (81,111 bp, 51.77%) followed by rRNA genes (9,086 bp, 5.80%) and tRNA genes (2,494 bp, 1.59%) (counting both IRs).

**Table 1:** Comparison of general features of fern chloroplast genomes

	*Alsophila spinulosa*	*Adiantum capillus-veneris*	*Psilotum nudum*	*Angiopteris evecta*
Total Length (bp)	156661	150568	138829	153901
GC content (%)	40.43	42.01	36.03	35.48
LSC Length (bp)	86308	82282	84617	89709
GC content (%)	39.62	40.82	33.63	33.66
SSC Length (bp)	21623	21392	16304	22086
GC content (%)	37.85	37.10	29.97	33.05
IR Length (bp)	24365	23447	18954	21053
GC content (%)	43.02	46.33	44.00	40.65
Number of gene^a^	117	117	118	121
Protein gene	85	84	81	85
rRNA gene	4	4	4	4
tRNA gene	28	29^b^	33	32

^a ^The genes in IRs were considered only once

^b ^*trnSeC *was counted here according to the annotation in GenBank and the reference [10]

The *Alsophila *cp genome has an overall GC content of 40.43%, which is lower only than *Adiantum capillus-veneris *amongst the four sequenced fern cp genomes (Table 1) and is the fourth highest amongst sequenced land plant cp genomes (see Additional file 1). Like other land plants [15,16], GC content is unevenly distributed across the *Alsophila *cp genome by location, functional group and codon position. The GC content in rRNA genes (55.18%) and tRNA genes (54.55%) is much higher than in protein coding regions (40.87%). The GC percentage in IRs is the highest (Table 1), reflecting the high GC content of rRNA genes. Amongst the protein genes, photosynthetic genes possess the highest GC content (43.85%), followed by genetic system genes (40.80%), whilst NADH genes have the least (39.54%). The GC content also varies by codon position with the first (47.75%) > second (40.94%) > third (33.91%) position in turn.

The start codons of 85 protein genes were inferred by comparisons with previously annotated land plant cp genomes. Sixty-three of these genes start with AUG, 20 with ACG and 2 with GUG (*psbC *and *rps12*). An ACG codon may be restored to a canonical start codon (AUG) by RNA editing, whereas a GUG initiation codon has been reported in other cp genomes [17,18]. Inferring translation start positions based only on genome sequences is merely hypothetical [10]. Future determination of sequences from complementary DNA (cDNA) and/or proteins will help to substantiate the putative translation start positions as well as RNA editing sites.

**Figure 2:**
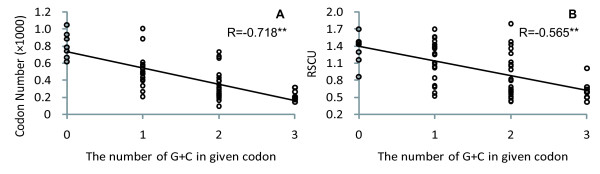
**Correlations between codon usage and codon GC content in the *Alsophila spinulosa *cp genome**. Codon usage is represented by total number (A) and RSCU (Relative Synonymous Codon Usage) (B) of each codon; codon GC content is indicated by the number of G+C in given codon. Each point represents one of the 59 degenerate codons. Pearson correlations shown in the figure are all significant at p < 0.001. A, correlation between total number and GC content of each codon; B, correlation between RSCU value and GC content of each codon.

There are in total 27,046 codons in all protein coding regions (including coding regions in both IRs) (Table 2), representing the total coding capacity of the *Alsophila *cp genome; of these, 2771 (10.25%) are for leucine, 2365 (8.74%) for serine, 2154 (7.96%) for isoleucine, and 1847 (6.83%) for glycine. One third of the total codons are represented by these four amino acids. The codon usage of the *Alsophila *cp genome reflects an apparent AT bias. Most codons end in A or U (66.13%). As shown in figure 2, both codon numbers and RSCU (Relative Synonymous Codon Usage) values are negatively correlated with codon GC content (represented by the number of G+C in a given codon). It appears that nucleotide composition bias has a significant influence on codon usage.

**Table 2:** Total numbers of each codon detected in all putative protein coding regions in the *Alsophila spinulosa *chloroplast genome, indicated with tRNAs for which genes have been identified

AA	Codon	Number	tRNA	AA	Codon	Number	tRNA
Phe	UUU	740		Ser	UCU	612	
Phe	UUC	539	trnF-GAA	Ser	UCC	390	trnS-GGA
Leu	UUA	787		Ser	UCA	499	trnS-UGA
Leu	UUG	577	trnL-CAA	Ser	UCG	268	
Tyr	UAU	669		Cys	UGU	204	
Tyr	UAC	267	trnY-GUA	Cys	UGC	91	trnC-GCA
Ter	UAA	82		ter	UGA	52	
Ter	UAG	27		Trp	UGG	435	trnW-CCA
Leu	CUU	471		Pro	CCU	389	
Leu	CUC	237		Pro	CCC	312	trnP-GGG
Leu	CUA	479	trnL-UAG	Pro	CCA	344	trnP-UGG
Leu	CUG	220		Pro	CCG	185	
His	CAU	407		Arg	CGU	397	trnR-ACG(×2)
His	CAC	192	trnH-GUG(×2)	Arg	CGC	144	
Gln	CAA	580	trnQ-UUG	Arg	CGA	314	trnR-UCG
Gln	CAG	247		Arg	CGG	170	
Ile	AUU	1051		Thr	ACU	530	
Ile	AUC	488	trnI-GAU(×2)	Thr	ACC	294	trnT-GGU
Ile	AUA	615	trnI-CAU	Thr	ACA	394	
Met	AUG	543	trnfM-CAU trnM-CAU	Thr	ACG	208	
Asn	AAU	938		Ser	AGU	431	
Asn	AAC	332	trnN-GUU(×2)	Ser	AGC	165	trnS-GCU
*Lys*	AAA	883		Arg	AGA	497	trnR-UCU
*Lys*	AAG	472		Arg	AGG	232	
Val	GUU	563		Ala	GCU	734	
Val	GUC	216		Ala	GCC	262	
Val	GUA	564	trnV-UAC	Ala	GCA	428	trnA-UGC(×2)
Val	GUG	236		Ala	GCG	204	
Asp	GAU	885		Gly	GGU	683	
Asp	GAC	241	trnD-GUC	Gly	GGC	191	trnG-GCC
Glu	GAA	1006	trnE-UUC	Gly	GGA	658	trnG-UCC
Glu	GAG	460		Gly	GGG	315	

### Gene order

**Figure 3:**
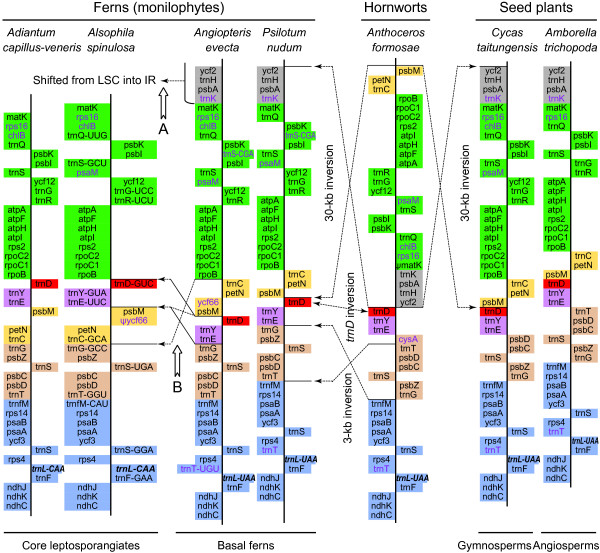
**Expected rearrangements in the evolution of fern cp genomes**. Genes are represented by boxes extending right or left of the base-line according to the direction of transcription. Each colored gene segment shows the same gene order region among the seven land plants cp genomes. The boxes highlighted in red denote the inversion of *trnD-GUC*. Excluding *Alsophila spinulosa*, the unchanged tRNA anti-codon is abbreviated in the other six cp genomes. The genes that are missing in one or more cp genomes are shown in purple. The tRNA-leu (CAA/UAA) gene between *rps4 *and *ndhJ *is indicated in bold italic type. The pseudogene is denoted by ψ. A, details are shown in the following Figure 5; B, hypothetical pathways to explain this rearrangement are illustrated in the following Figure 6.

The *Alsophila *cp genome shares three key inversions with other ferns relative to bryophytes (Figure 3): 1) a 30-kb inversion at the beginning of LSC (close to IRA) [2]; 2) an approximately 3 kb inversion involving *trnT*, *psbD*, *psbC*, *trnS*, *psbZ *and *trnG *[8,10,19]; and 3) a minor inversion containing a single gene *trnD-GUC*. The first of these inversions is also shared by all vascular plants except lycophytes, whereas the latter two are restricted to ferns.

To our knowledge, the *trnD-GUC *inversion has not been previously documented. Three conserved and consecutive tRNA genes, *trnD-GUC*, *trnY-GUA *and *trnE-UUC*, have been identified in all land plant cp genomes. Excluding ferns, the three genes have the same directions of transcription. However, in ferns *trnD *is inverted relative to *trnY *and *trnE *(Figure 3). The simplest interpretation of this change is a single minor inversion involving only *trnD*. Based on current data, it remains unknown whether the 3-kb inversion or the *trnD *inversion occurred first in ferns.

**Figure 4:**
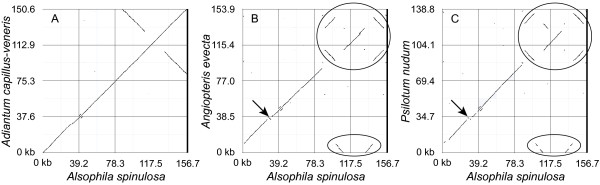
**Comparisons of the gene order of *Alsophila spinulosa *cp genome with other ferns**. Comparison by using online zPicture software [44]. Points along the positive slope are in the same orientation in both genomes, whereas points along the negative slope indicate sequences that are oriented in opposite directions. A, the two groups of points that fall along the negative slope in the upper right corner represent the sequences of IRs. B and C, ellipses denote the complex rearrangements in IRs and arrows indicate the gene order change between *rpoB *and *psbZ*.

**Figure 5:**
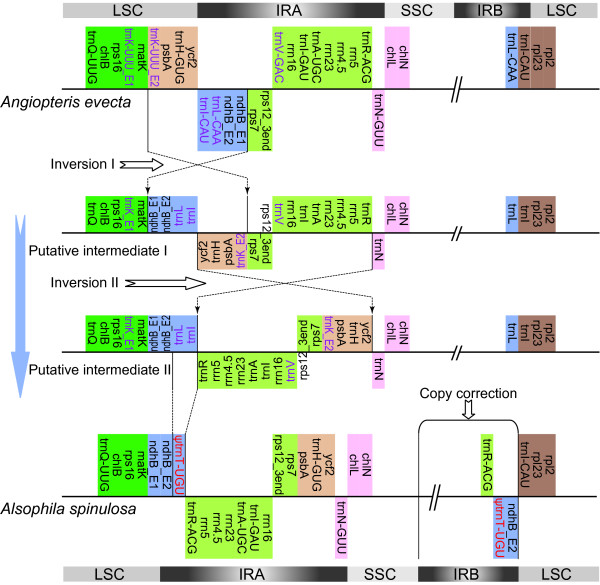
**The "two inversions" model for IR rearrangements in fern cp genomes**. Genes are represented by boxes extending above or below the base-line according to the direction of transcription. The genes that are absent in the *Alsophila *cp genomes are shown in purple: these loci are merely hypothetical in putative intermediates since the course of their loss is unclear. The pseudogene of *trnT-UGU *is represented in red and indicated by ψ. The tRNA anti-codon is abbreviated in putative intermediates.

Overall, the *Alsophila *cp genome shows a high degree of synteny with the previously sequenced cp genome of *Adiantum *(Figure 4A). In contrast, there exist striking differences between *Alsophila *and *Angiopteris *(Figure 4B) as well as between *Alsophila *and *Psilotum *(Figure 4C). A set of complex rearrangements in the IRs, involving a rare duplication of *psbA *gene, was found in "higher" ferns using physical mapping [3,4]. The IR gene orders of "higher" ferns, such as *Adiantum*, *Cyathea *and *Polystichum*, are highly rearranged in comparison to that of basal leptosporangiate *Osmunda *[3,4,20]. Complete cp genome data from *Angiopteris*, *Psilotum*, *Adiantum *and *Alsophila *detail these rearrangements. The IR gene order in *Alsophila *appears to be the same as that in *Adiantum*, while *Angiopteris *and *Psilotum *have the *Osmunda *gene order. To explain the complex rearrangements, a "two inversions" hypothesis was proposed [20]. Figure 5 illustrates the great gene order changes within these rearrangements and the updated version of the "two inversions" model incorporating gene order data from the *Alsophila *and *Angiopteris *cp genomes. Recently, Wolf and Roper [9] indicated that the two major inversions did occur in turn and the second inversion (Figure 5, Inversion II) took place on the branch leading to the common ancestor of the heterosporous fern clade and its sister group. Thus, it seems reasonable to hypothesize that the *Adiantum *gene order represents a common feature of the three lineages within core leptosporangiates (including heterosporous ferns, tree ferns and polypod ferns).

Interestingly, in the *Adiantum *cp genome, an intron-containing *trnT-UGU *was identified between *trnR-ACG *and *ndhB *(Table 3) [10]. The *Alsophila *cp genome possesses no intact intron-containing *trnT*. However, two fragments that are similar to the two exons within the *Adiantum trnT *were annotated as a *ΨtrnT-UGU *in this study (Table 3). This new *trnT *or *ΨtrnT *is just at one endpoint of the Inversion II (Figure 5). Therefore, the generation of intron-containing *trnT-UGU *may be associated with the IR rearrangements.

**Table 3:** Comparison of gene contents of fern chloroplast genomes

Gene^a^	*Alsophila spinulosa*	*Adiantum capillus-veneris*	*Psilotum nudum*	*Angiopteris evecta*
tRNA gene				
*trnR-CCG *(*rbcL-accD*)	*trnR-UCG*	*trnSeC*	●	●
*trnL-CAA *(*ndhB *Exon2 3')	○	○	●	●
*trnL-UAA *(*rps4-ndhJ*)	*trnL-CAA*	*trnL-CAA*	●	●
*trnK-UUU *(*MatK*)	○	○	●	●
*trnS-CGA *(*psbK-psbI*)	○	○	●	●
*trnT-UGU *(*rps4-ndhJ*) (without intron)	○	○	●	●
*trnT-UGU *(*ndhB *Exon2 3') (with one intron)	▲	●	○	○
*trnV-GAC *(*rrn16-rps12*)	○	○	●	●
*trnG-UCC *(*ycf12-atpA*)	●	●	●	○
Protein gene				
*psaM*	●	○	●	●
*ycf66*	▲	○	○	●
*chlB*	●	●	○	●
*chlL*	●	●	○	●
*chlN*	●	●	○	●
*rps16*	●	●	○	●
*ycf1*	●	●	●	▲

^a ^Only the genes that are missing in one or more cp genomes are shown. The loci of tRNA genes are denoted by their neighbor protein genes. A filled/open circle denotes the presence/absence of a gene. A filled triangle indicates a pseudogene.

**Figure 6:**
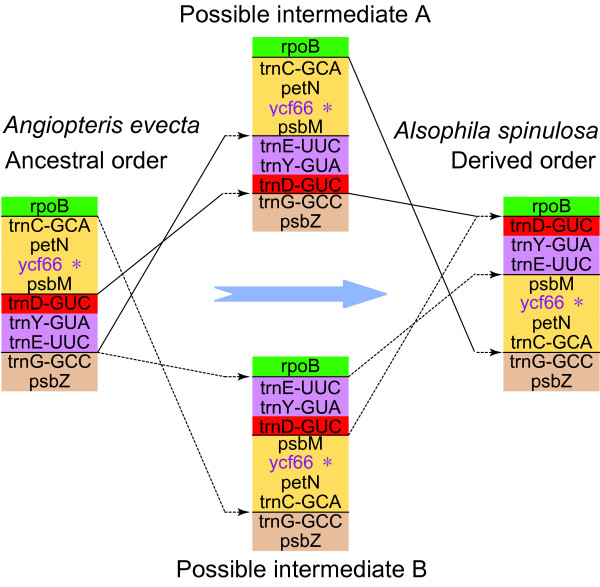
**Two hypothetical pathways to explain rearrangement between *rpoB *and *psbZ***. Genes are represented by boxes, the colors of which are consistent with Figure 3. *In *Angiopteris evecta ycf66 *has an intact ORF, but in *Alsophila spinulosa *it is a pseudogene.

*Alsophila *and *Adiantum *share another rearranged region between *rpoB *and *psbZ *in LSC relative to *Angiopteris *and *Psilotum *(Figure 3). For the latter two, gene order in this region is "***rpoB***-*trnC*-*petN*-*psbM*-*trnD*-*trnY*-*trnE-****trnG***-***psbZ***", whereas in *Alsophila *and *Adiantum *it is "***rpoB***-*trnD*-*trnY*-*trnE*-*psbM*-*petN*-*trnC*-***trnG***-***psbZ***" (Genes with boldface are unchanged) (Figure 3). Roper *et al*. [8] noted that this gene order change is not caused by a single inversion. Two alternative pathways may account for this rearrangement (Figure 6), but more data are needed to determine the order of the two inversions.

### Gene content

A total of 117 different genes are present in the *Alsophila *cp genome (Table 1). This gene content is similar to that of most land plants [21]. However, there are some interesting differences amongst the four sequenced fern cp genomes (Table 3). The *Alsophila *cp genome possesses the least number of tRNA genes due to 5 missing tRNA genes in comparison to basal ferns (*Psilotum nudum *and *Angiopteris evecta*). Its protein gene number is equal to that of *Angiopteris*, but higher than that of both *Adiantum *and *Psilotum*. Details of these differences are discussed below.

#### Novel tRNA gene

A unique *trnR-UCG *gene, encoding tRNA-Arg, is found between *rbcL *and *accD *in the *Alsophila *cp genome (Figure 1; Table 3). Another type of tRNA-Arg gene *trnR-CCG *resides in the same locus in non-flowering land plants including *Angiopteris *[8] and *Psilotum *[19]. In *Adiantum*, an apparent tRNA gene is annotated as *trnSeC *[10]. It is uncertain whether the occurrence of *trnR-UCG *in the *Alsophila *cp genome represents a unique feature for this species or is an apomorphy for a larger clade such as Cyatheaceae or tree ferns. To address this question, we collected all fern *rbcL*-*accD *intergenic sequences deposited in GenBank and examined the tRNA genes within them using ARAGORN [22]. The results indicate that *trnR-UCG *is restricted to tree ferns, whereas *trnR-CCG *is widespread in non-core leptosporangiates and basal ferns (Table 4). However, neither *trnR-UCG *nor the *trnR-CCG *gene is identified at this locus in polypod ferns. Therefore, the existence of *trnR-UCG *may reflect a putative molecular apomorphy of tree ferns.

**Table 4:** tRNA genes in fern *rbcL*-*accD *intergenic spacer sequences^a^

Order^b^	Family	Number of sequences	tRNA gene^c^
Cyatheales (tree ferns)	Cyatheaceae	140	*trnR-UCG*
	Dicksoniaceae	6	*trnR-UCG*
	Lophosoriaceae	1	*trnR-UCG*
	Hymenophyllopsidaceae	1	*trnR-UCG*
Gleicheniales	Dipteridaceae	1	*trnR-CCG*
	Gleicheniaceae	2	*trnR-CCG*
Hymenophyllales	Hymenophyllaceae	84	*trnR-CCG*
Osmundales	Osmundaceae	18	*trnR-CCG*
Marattiales	Marattiaceae	1	*trnR-CCG*
Psilotales	Psilotaceae	1	*trnR-CCG*
Ophioglossales	Ophioglossaceae	1	*trnR-CCG*

^a ^All sequence data were obtained from GenBank at March 30, 2009;

^b ^Group names at ordinal level follow Smith et al. [7];

^c ^tRNA genes were identified by using ARAGORN v1.2 [22]

**Figure 7:**
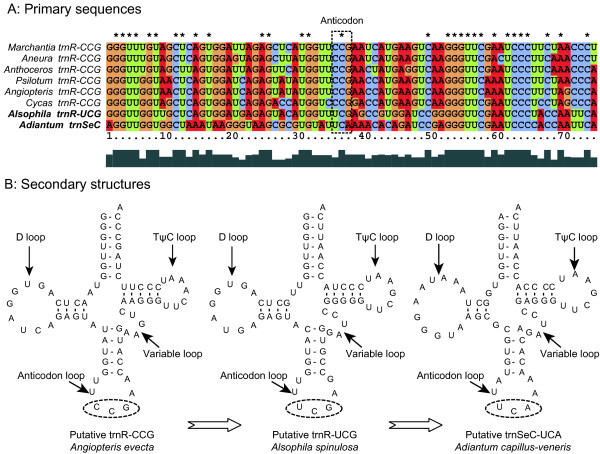
**Comparisons of the sequences and putative secondary structures of trnR-CCG, trnR-UCG and trnSeC (tRNA-selenocysteine)**. A, primary sequences of *Alsophila spinulosa *trnR-UCG, *Adiantum capillus-veneris *trnSeC and trnR-CCGs from other six land plants. Dashed rectangle indicates anticodons. B, the putative secondary structures of *Angiopteris evecta *trnR-CCG,*Alsophila *trnR-UCG and *Adiantum *trnSeC. Dashed ellipses indicate anticodons.

Sequence alignment indicates that trnR-UCG and trnR-CCGs have quite similar primary sequences with 44 of 74 nucleotides unchanged across 7 representative land plants (Figure 7A). In addition, the *Adiantum *trnSeC shares 51, 41 and 40 identical nucleotides with the *Alsophila *trnR-UCG, the *Psilotum *trnR-CCG and the *Angiopteris *trnR-CCG respectively (Figure 7A). Due to their similarities and conserved loci, we propose that *Alsophila trnR-UCG*, *Adiantum trnSeC *as well as *trnR-CCG*s in other land plants are orthologous. Tree fern trnR-UCG can transfer arginine even though its anticodon alters from CCG to UCG. However, *Adiantum *trnSeC has undergone major changes: 1) its anticodon is UCA (unmatchable for an Arg codon), and 2) it contains up to 18 nucleotide differences relative to all other land plant trnR genes (Figure 7A). Our findings imply that the *trnR-UCG *is derived from the *trnR-CCG *by the alteration of one anticodon base; then the *Adiantum trnSeC *evolves from the *trnR-UCG *by altering one anticodon base further, becoming a *trnR-UCG *pseudogene (Figure 7B). If this is the case, the *Adiantum trnSeC *should be annotated as *ΨtrnR*. Sugiura and Sugita [23] argued that the *trnR-CCG *is not essential for plastid function although it is conserved in non-flowering plants. The evolutionary scenario of *trnR-CCG *in ferns (Figure 7B) tends to support this view.

At the locus between *rps4 *and *ndhJ*, the *Alsophila *and *Adiantum *cp genomes encode a *trnL-CAA *(tRNA-Leu) rather than a *trnL-UAA *gene (Table 3). However, they lose another *trnL-CAA *gene (Table 3), which is found at the 3' downstream of *ndhB *in almost all other land plant plastid genomes. Consequently, *Alsophila *and *Adiantum *only possess the *trnL-CAA*, whereas the *Angiopteris *and *Psilotum *cp genomes contain both the *trnL-UAA *and the *trnL-CAA*. In the *Adiantum *chloroplast, the missing trnL-UAA could be provided for the heavily used UUA codon by a partial C-to-U edit in the trnL-CAA anticodon [24]. Since the UUA is also a preferred leucine codon for the *Alsophila *cp genome (RSCU = 1.70), the same editing event might occur in the *Alsophila *chloroplast as well.

#### Missing tRNA gene

Only 28 tRNA genes are encoded in the *Alsophila *cp genome, whereas 29, 32 and 33 are annotated in *Adiantum*, *Angiopteris *and *Psilotum*, respectively (Table 1). For cp genomes, it is believed that a set of 30 tRNA species is sufficient for the translation of chloroplast mRNAs [25]. In the *Angiopteris *and *Psilotum *chloroplasts, tRNAs can read all codons by using two-out-of-three and wobble mechanisms [26]. However, in *Alsophila *and *Adiantum *chloroplasts, both lysine codons lack a corresponding tRNA-Lys (encoded by *trnK*) (Table 2; Table 3). The loss of *trnK *suggests cytosolic tRNAs may be imported into chloroplasts, despite a lack of experimental evidence [27]. As an incidental consequence of the *trnK *loss, the *matk *open reading frame (ORF) is not nested in the *trnK *intron (Figure 1).

Apart from the *trnK *and the *trnL-CAA*, the *Alsophila *cp genome also shares other 3 tRNA gene losses, including the *trnS-CGA*, the *trnV-GAC *and the *trnT-UGU *(intron-free), with *Adiantum *relative to basal ferns *Angiopteris *and *Psilotum *(Table 3). The shared absence of tRNA genes between *Alsophila *and *Adiantum *suggests that they may derive from a common ancestor.

#### Protein genes

The *Alsophila *cp genome contains a *psaM *gene encoding photosystem I reaction center subunit M. This gene has been detected in *Psilotum *[19] and *Angiopteris *[8], but not in *Adiantum *[10] (Table 3). Besides ferns, *psaM *also exists in bryophytes, lycophytes and gymnosperms, but not in angiosperms, implying its independent loss in ferns and angiosperms [10]. *Alsophila *and *Adiantum *represent tree ferns and polypods, respectively. Due to their sister relationship, we speculate that the loss of *psaM *in ferns occurred after the split of polypods and tree ferns.

A putative pseudogene of *ycf66 *is identified in the *Alsophila *cp genome (Figure 1; Table 3). The 5' ends of its two exons are both destroyed. In the four sequenced fern cp genomes, only *Angiopteris *contains an intact *ycf66 *gene [8]. For other land plants, this gene only occurs in *Marchantia polymorpha *(liverworts), *Physcomitrella patens *subsp.*patens *(mosses),*Syntrichia ruralis *(mosses) and *Huperzia lucidula *(lycophytes). The findings suggest that *ycf66 *is lost independently in multiple clades of land plants including hornworts, ferns and seed plants.

### Inversion Endpoint as Hotspot for Repeats

**Figure 8:**
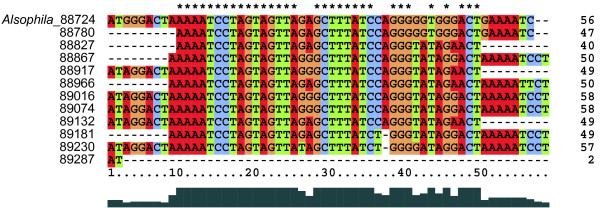
**Repetitive units within the highly repeated 565-bp sequence**. The sequence from 88,724 to 89,288 bp in IRB is shown in this figure. The numbers on the right hand side indicate the lengths of each segment.

A total of 133 pairs of repeats (≥ 30 bp) were identified in the *Alsophila *cp genome by using REPuter [28], of which 106 are direct and 27 are inverted repeats. This number of repeats is less than are found in some highly rearranged cp genomes (e.g. *Trachelium caeruleum*) but more than are present in unrearranged ones (e.g. *Nicotiana tabacum*) [29,30]. Up to 66 direct repeats (no inverted repeat) are restricted to a region spanning only 565 bp (153,682–154,246 bp in IRA or 88,724–89,288 bp in IRB) between *trnR-ACG *and *ψtrnT-UGU *in the IRs (Figure 1). The GC content of this 565-bp-region (35.93%) is lower than that of IRs and the overall GC content of the whole genome. Detailed sequence analyses revealed that this region is composed of tandem iterations of 11 similar segments ranging from 40 to 58 bp (Figure 8). The core repeated motif is AAAATCCTAGTAGTTAgaGCTTTATCcaGGGtaTaGgACT (the lowercase letters denote variable bases) with variant lengths of heads and/or tails (Figure 8).

**Figure 9:**
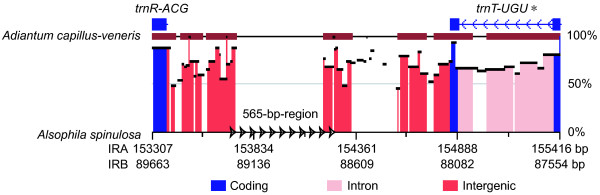
**Alignment of the sequence from *trnR-ACG *to *ψtrnT-UGU *in *Alsophila *and corresponding region in *Adiantum***. Comparison by using online zPicture software with ECR criteria of ≥ 100 bp and ≥ 70% identity [44]. Similarities between aligned regions are shown as average percent identity. The cluster of arrows denotes the location of the highly repeated 565-bp-region in the *Alsophila *cp genome. *, an intact *trnT-UGU *was identified in *Adiantum*, while a *trnT-UGU *pseudogene was found in *Alsophila*.

In contrast to *Alsophila*, dispersed repeats (≥ 30 bp) are rare in the *Adiantum *cp genome, with only 5 short inverted repeats (30–36 bp); and none of these occurs between the *trnR-ACG *and the *trnT-UGU*. In the *Alsophila *cp genome, the length of the intergenic region between *trnR-ACG *and *ψtrnT-UGU *is 1467 bp, whereas in *Adiantum *it is 913 bp, the difference being 554 bp. We noted that this length is very similar to that of the highly repeated 565-bp-region, and speculate that the difference is caused by the presence of the highly repeated region. To test this hypothesis, we extracted the sequence from *trnR-ACG *to *ψtrnT-UGU *in *Alsophila *and from *trnR-ACG *to *trnT-UGU *in *Adiantum*. The sequence alignment indicates that the highly repeated 565-bp-region is indeed lost in the *Adiantum *cp genome (Figure 9).

In the *Alsophila *cp genome, the location of the highly repeated 565-bp-region is exactly at the endpoint of the second inversion of the IR rearrangements (Figure 5, Inversion II). Dispersed repeated sequences have been reported from several cp genomes. These are associated with numerous DNA rearrangements, particularly inversions [31-33]. In extensively rearranged cp genomes, the endpoints of rearranged gene clusters are usually flanked by repeated sequences [29,30,34]. If repeat-mediated recombination is the major mechanism generating inversions in cp genomes [35,36], the preservation of repeats would destabilize the genome structure. After inversions, the repeats should be deleted to guarantee genome stability (like the situation in *Adiantum*). The repeats observed at the endpoint of the ancient inversion (Figure 5, Inversion II) may be a vestige of recent rearrangement(s) that are undiscovered. The existence of these repeats implies that the region is a potential hotspot for genomic reconfiguration.

## Conclusion

In this study, we present the first complete cp genome sequence from a tree fern and provide a comprehensive comparative analysis of cp genomes in ferns. The cp genome size of *Alsophila *is larger than that of *Adiantum, Psilotum *and *Angiopteris*. Besides 117 genes, two pseudogenes *Ψycf66 *and *ΨtrnT-UGU *are also detected in the *Alsophila *cp genome. An intact *ycf66 *is identified in *Angiopteris*, while an intron-containing *trnT-UGU *is found in *Adiantum*. Based on the findings, we speculate that *Ψycf66 *reflects an intermediate during *ycf66 *gene loss, and the genesis of *trnT-UGU *may be associated with the IR rearrangements. A *trnR-UCG *gene was detected between *rbcL *and *accD *in *Alsophila*, and this seems to be a molecular apomorphy of tree ferns. In the *Adiantum *cp genome, the *trnR-UCG *gene degenerates to a pseudogene. The *Alsophila *cp genome shares several unusual characteristics with the previously sequenced *Adiantum *(a polypod fern) cp genome, such as five missing tRNA genes and two major rearranged regions. These common characters probably derive from their common ancestor. In the *Alsophila *cp genome, a highly repeated 565-bp-region, which is composed of tandem iterations of 11 similar segments, occurs at one endpoint of an ancient inversion, but it is not detected in the genome of *Adiantum*. Nonetheless, the origin and function of these repeats remain to be characterized in future studies.

## Methods

### Genome sequencing and assembly

Young leaves of *Alsophila spinulosa *were collected from a plant growing in the greenhouse in Wuhan Botanical Garden, Chinese Academy of Sciences. A voucher specimen was deposited at Wuhan Botanical Garden. Total DNA was extracted using the CTAB-based method [37]. The cp genome was amplified using polymerase chain reaction (PCR). In brief, the coding sequences were extracted from known chloroplast genomic sequences of three ferns [GenBank: NC_003386, NC_008829 and NC_004766], three bryophytes [GenBank: NC_001319, NC_005087 and NC_004543] and one lycophyte [GenBank: NC_006861] according to their annotations in GenBank. PCR primers were developed from alignments of the above coding sequences. Overlapping regions of each pair of adjacent PCR fragments exceeded 150 bp. We did not clone two inverted repeats (IRs) separately, but designed primers to amplify the regions spanning the junctions of LSC/IRA, LSC/IRB, SSC/IRA and SSC/IRB. Using these primers, we covered the entire cp genome of *Alsophila *with PCR products ranging in size from 500 bp to 5 kb. All PCR reactions were performed using TaKaRa LA taq (TaKaRa Bio Inc, Shiga, Japan). Amplified cp genome fragments were cloned into TaKaRa pMD19-T plasmids (TaKaRa Bio Inc, Shiga, Japan), which were then used to transform *E. coli *DH5α. Multiple (≥ 6) clones were randomly selected and commercially sequenced using ABI 3730xl DNA Analyzer (Applied Biosystems, Foster City, CA). For long fragments (> 1.4 kb), walking primers were designed based on acquired sequences and used for sequencing remaining sequences step by step. Gap regions (caused by unsuccessful PCR amplification or failed primer walking sequencing) were amplified using primers that flank the gaps, then cloned and sequenced as above. From the individual reads we excluded vector, primer and low-quality sequences, then we assembled the reads using Phrap [38]. Since automated assembly methods cannot distinguish two IRs, we input the reads as two parts and acquired two large contigs, with each contig including one IR and its adjacent partial large and small single copy (LSC and SSC) regions. Then the two large contigs were manually assembled into the complete circular genome sequence. Inverted repeats were identified through alignment of the final complete genome sequence against itself via Blast 2 sequences at the National Center for Biotechnology Information [39]. We accumulated 1,415,559 bp sequences, which is about 9-fold coverage.

### Annotation and related study

Annotation of the *Alsophila *cp genome was performed using DOGMA (Dual Organellar GenoMe Annotator) [40]. Genes that were undetected by DOGMA, such as *ycf1*, *ycf2*, *rps16*, *ndhF*, *ndhG *and *matK*, were identified by Blastx . From this initial annotation, putative starts, stops, and intron positions were determined by comparisons with homologous genes in other cp genomes and by considering the possibility of RNA editing, which can modify the start and stop positions. tRNA genes were annotated using DOGMA and ARAGORN v1.2 [22], and then confirmed by ERPIN [41] and TFAM Webserver v1.3 [42]. The circular gene map of the *Alsophila *cp genome was drawn by GenomeVx [43] followed by manual modification.

Synteny among fern cp genomes was analyzed and visualized by using online zPicture software [44].

### Examination of GC content

Overall GC content was calculated for 118 land plant plastid genomes (see Additional file 1). For the *Alsophila *cp genome, GC content was farther determined for three groups of genes, protein-coding genes (85), rRNA genes (4) and tRNA genes (28), respectively. For protein-coding genes, GC content was calculated for the entire gene and the first, second and third codon positions, respectively. Protein-coding genes were partitioned into three main functional groups: photosynthetic genes, genetic system genes and NADH genes. GC content of the three groups of genes was then determined. The genes included in each of these three groups were: (1) photosynthetic genes (*rbcL*, atp*, pet*, psa* and psb*); (2) genetic system genes (rpl*, rps*, rpo*, *clpP*, *infA *and *matK*); and (3) NADH genes (ndh*).

### Dispersed repeats

Direct and inverted repeats in the *Alsophila *and *Adiantum *[GenBank: NC_004766] cp genomes were determined by using REPuter [28] at a repeat length ≥ 30 bp with a Hamming distance of 3. The entire genome was used to detect repeats in order to map them in both copies of the IR, but numbers of repeats were based on results from only one IR copy.

## List of abbreviations

cp: chloroplast; IR: inverted repeat; LSC: large single copy; SSC: small single copy; RSCU: relative synonymous codon usage.

## Authors' contributions

LG participated in the conception of this study, carried out part of the genome sequencing, performed all sequence analyses, annotated the genome, generated tables and figures, and drafted the manuscript. XY and YXY participated in genome sequencing. YJS and TW conceived and supervised the project, contributed to the interpretation of the data and prepared the manuscript. All authors read and approved the final manuscript.

## Supplementary Material

Additional file 1 **Additional Table 1**. a list of published complete chloroplast sequences in land plants. Click here for file

## References

[B1] Raubeson LA, Jansen RK, Henry RJ (2005). Chloroplast genomes of plants. Plant diversity and evolution: genotypic and phenotypic variation in higher plants.

[B2] Raubeson LA, Jansen RK (1992). Chloroplast DNA evidence on the ancient evolutionary split in vascular land plants. Science.

[B3] Stein DB, Conant DS, Ahearn ME, Jordan ET, Kirch SA, Hasebe M, Iwatsuki K, Tan MK, Thomson JA (1992). Structural rearrangements of the chloroplast genome provide an important phylogenetic link in ferns. Proc Natl Acad Sci USA.

[B4] Raubeson LA, Stein DB (1995). Insights into fern evolution from mapping chloroplast genomes. Am Fern J.

[B5] Pryer KM, Schneider H, Zimmer EA, Ann Banks J (2002). Deciding among green plants for whole genome studies. Trends Plant Sci.

[B6] Schneider H, Schuettpelz E, Pryer KM, Cranfill R, Magallon S, Lupia R (2004). Ferns diversified in the shadow of angiosperms. Nature.

[B7] Smith AR, Pryer KM, Schuettpelz E, Korall P, Schneider H, Wolf PG (2006). A classification for extant ferns. Taxon.

[B8] Roper JM, Kellon Hansen S, Wolf PG, Karol KG, Mandoli DF, Everett KDE, Kuehl J, Boore JL (2007). The complete plastid genome sequence of *Angiopteris evecta *(G. Forst.) Hoffm. (Marattiaceae). Am Fern J.

[B9] Wolf PG, Roper JM, Ranker TA, Haufler CH (2008). Structure and evolution of fern plastid genomes. Biology and evolution of ferns and lycophytes.

[B10] Wolf PG, Rowe CA, Sinclair RB, Hasebe M (2003). Complete nucleotide sequence of the chloroplast genome from a leptosporangiate fern, *Adiantum capillus-veneris *L. DNA Res.

[B11] Pryer KM, Schuettpelz E, Wolf PG, Schneider H, Smith AR, Cranfill R (2004). Phylogeny and evolution of ferns (monilophytes) with a focus on the early leptosporangiate divergences. Am J Bot.

[B12] Pryer KM, Schneider H, Smith AR, Cranfill R, Wolf PG, Hunt JS, Sipes SD (2001). Horsetails and ferns are a monophyletic group and the closest living relatives to seed plants. Nature.

[B13] Janssen T, Bystriakova N, Rakotondrainibe F, Coomes D, Labat JN, Schneider H (2008). Neoendemism in Madagascan scaly tree ferns results from recent, coincident diversification bursts. Evolution.

[B14] Conant DS, Stein DB, Valinski AEC, Sudarsanam P, Ahearn ME (1994). Phylogenetic implications of chloroplast DNA variation in the Cyatheaceae. I. Syst Bot.

[B15] Cai Z, Penaflor C, Kuehl JV, Leebens-Mack J, Carlson JE, dePamphilis CW, Boore JL, Jansen RK (2006). Complete plastid genome sequences of *Drimys*, *Liriodendron*, and *Piper*: implications for the phylogenetic relationships of magnoliids. BMC Evol Biol.

[B16] Shimada H, Sugiura M (1991). Fine structural features of the chloroplast genome: comparison of the sequenced chloroplast genomes. Nucleic Acids Res.

[B17] Sugiura M, Hirose T, Sugita M (1998). Evolution and mechanism of translation in chloroplasts. Annu Rev Genet.

[B18] Kuroda H, Suzuki H, Kusumegi T, Hirose T, Yukawa Y, Sugiura M (2007). Translation of *psbC *mRNAs starts from the downstream GUG, not the upstream AUG, and requires the extended shine-dalgarno sequence in tobacco chloroplasts. Plant Cell Physiol.

[B19] Wakasugi T, Nishikawa A, Yamada K, Sugiura M (1998). Complete nucleotide sequence of the plastid genome from a fern, *Psilotum nudum*. Endocyt Cell Res.

[B20] Hasebe M, Iwatsuki K (1992). Gene localization on the chloroplast DNA of the maiden hair fern; *Adiantum capillus-veneris*. J Plant Res.

[B21] Wakasugi T, Tsudzuki T, Sugiura M (2001). The genomics of land plant chloroplasts: Gene content and alteration of genomic information by RNA editing. Photosynth Res.

[B22] Laslett D, Canback B (2004). ARAGORN, a program to detect tRNA genes and tmRNA genes in nucleotide sequences. Nucleic Acids Res.

[B23] Sugiura C, Sugita M (2004). Plastid transformation reveals that moss tRNA^Arg^-CCG is not essential for plastid function. Plant J.

[B24] Wolf PG, Rowe CA, Hasebe M (2004). High levels of RNA editing in a vascular plant chloroplast genome: analysis of transcripts from the fern *Adiantum capillus-veneris*. Gene.

[B25] Shinozaki K, Ohme M, Tanaka M, Wakasugi T, Hayashida N, Matsubayashi T, Zaita N, Chunwongse J, Obokata J, Yamaguchi-Shinozaki K (1986). The complete nucleotide sequence of the tobacco chloroplast genome: its gene organization and expression. EMBO J.

[B26] Pfitzinger H, Weil JH, Pillay DT, Guillemaut P (1990). Codon recognition mechanisms in plant chloroplasts. Plant Mol Biol.

[B27] Lung B, Zemann A, Madej MJ, Schuelke M, Techritz S, Ruf S, Bock R, Huttenhofer A (2006). Identification of small non-coding RNAs from mitochondria and chloroplasts. Nucleic Acids Res.

[B28] Kurtz S, Choudhuri JV, Ohlebusch E, Schleiermacher C, Stoye J, Giegerich R (2001). REPuter: the manifold applications of repeat analysis on a genomic scale. Nucleic Acids Res.

[B29] Chumley TW, Palmer JD, Mower JP, Fourcade HM, Calie PJ, Boore JL, Jansen RK (2006). The complete chloroplast genome sequence of *Pelargonium × hortorum*: organization and evolution of the largest and most highly rearranged chloroplast genome of land plants. Mol Biol Evol.

[B30] Haberle RC, Fourcade HM, Boore JL, Jansen RK (2008). Extensive rearrangements in the chloroplast genome of *Trachelium caeruleum *are associated with repeats and tRNA genes. J Mol Evol.

[B31] Ogihara Y, Terachi T, Sasakuma T (1988). Intramolecular recombination of chloroplast genome mediated by short direct-repeat sequences in wheat species. Proc Natl Acad Sci USA.

[B32] Pombert JF, Lemieux C, Turmel M (2006). The complete chloroplast DNA sequence of the green alga *Oltmannsiellopsis viridis *reveals a distinctive quadripartite architecture in the chloroplast genome of early diverging ulvophytes. BMC Biol.

[B33] Pombert JF, Otis C, Lemieux C, Turmel M (2005). The chloroplast genome sequence of the green alga *Pseudendoclonium akinetum *(Ulvophyceae) reveals unusual structural features and new insights into the branching order of chlorophyte lineages. Mol Biol Evol.

[B34] Cai Z, Guisinger M, Kim HG, Ruck E, Blazier JC, McMurtry V, Kuehl JV, Boore J, Jansen RK (2008). Extensive reorganization of the plastid genome of *Trifolium subterraneum *(Fabaceae) is associated with numerous repeated sequences and novel DNA insertions. J Mol Evol.

[B35] Palmer JD (1985). Comparative organization of chloroplast genomes. Annu Rev Genet.

[B36] Palmer JD, Nugent JM, Herbon LA (1987). Unusual structure of geranium chloroplast DNA: A triple-sized inverted repeat, extensive gene duplications, multiple inversions, and two repeat families. Proc Natl Acad Sci USA.

[B37] Gawel N, Jarret R (1991). A modified CTAB DNA extraction procedure for *Musa *and *Ipomoea*. Plant Mol Biol Rep.

[B38] Ewing B, Green P (1998). Base-calling of automated sequencer traces using phred. II. Error probabilities. Genome Res.

[B39] Tatusova TA, Madden TL (1999). BLAST 2 Sequences, a new tool for comparing protein and nucleotide sequences. FEMS Microbiol Lett.

[B40] Wyman SK, Jansen RK, Boore JL (2004). Automatic annotation of organellar genomes with DOGMA. Bioinformatics.

[B41] Gautheret D, Lambert A (2001). Direct RNA motif definition and identification from multiple sequence alignments using secondary structure profiles. J Mol Biol.

[B42] Taquist H, Cui Y, Ardell DH (2007). TFAM 1.0: an online tRNA function classifier. Nucleic Acids Res.

[B43] Conant GC, Wolfe KH (2008). GenomeVx: simple web-based creation of editable circular chromosome maps. Bioinformatics.

[B44] Ovcharenko I, Loots GG, Hardison RC, Miller W, Stubbs L (2004). zPicture: dynamic alignment and visualization tool for analyzing conservation profiles. Genome Res.

