# Supplementary material for: Complete chloroplast genome sequence of a tree fern Alsophila spinulosa: insights into evolutionary changes in fern chloroplast genomes
Source: BMC Evol Biol. 2009 Jun 11;9:130. doi: 10.1186/1471-2148-9-130 (PMC2706227; doi:10.1186/1471-2148-9-130)
Supplement: Additional file 1 — Additional Table 1. a list of published complete chloroplast sequences in land plants. [file 1471-2148-9-130-S1.pdf]

Additional Table 1

List of published complete chloroplast sequences in land plants[a]

Family	Species	GenBank Accession Number	Genome size (bp)	GC content (%)
**Liverworts :**				
Marchantiaceae	*Marchantia polymorpha*	NC_001319	121024	28.81
Aneuraceae	*Aneura mirabilis*	NC_010359	108007	40.62
**Mosses:**				
Funariaceae	*Physcomitrella patens subsp. patens*	NC_005087	122890	28.53
Pottiaceae	*Syntrichia ruralis*	NC_012052	122630	28.37
**Hornworts:**				
Anthocerotaceae	*Anthoceros formosae*	NC_004543	161162	32.90
**Lycophytes:**				
Lycopodiaceae	*Huperzia lucidula*	NC_006861	154373	36.25
Selaginellaceae	*Selaginella uncinata*	AB197035	144170	54.85
**Monilophytes (ferns):**				
Psilotaceae	*Psilotum nudum*	NC_003386	138829	36.03
Marattiaceae	*Angiopteris evecta*	NC_008829	153901	35.48
**Cyatheaceae**	***Alsophila spinulosa***	**FJ556581**	**156661**	**40.43**
Pteridaceae	*Adiantum capillus-veneris*	NC_004766	150568	42.01

**Gymnosperms:**				
Cycadaceae	*Cycas taitungensis*	NC_009618	163403	39.45
Welwitschiaceae	*Welwitschia mirabilis*	NC_010654	119726	36.73
Ephedraceae	*Ephedra equisetina*	NC_011954	109518	36.64
Gnetaceae	*Gnetum parvifolium*	NC_011942	114914	38.18
Pinaceae	*Pinus thunbergii*	NC_001631	119707	38.50
Pinaceae	*Pinus koraiensis*	NC_004677	117190	38.80
Pinaceae	*Picea sitchensis*	NC_011152	109798	39.03
Pinaceae	*Pinus contorta*	NC_011153	115615	38.39
Pinaceae	*Pinus gerardiana*	NC_011154	116997	38.80
Pinaceae	*Pinus krempfii*	NC_011155	115555	38.73
Pinaceae	*Pinus lambertiana*	NC_011156	112521	38.45
Pinaceae	*Pinus longaeva*	NC_011157	115896	38.76
Pinaceae	*Pinus monophylla*	NC_011158	114607	38.70
Pinaceae	*Pinus nelsonii*	NC_011159	110575	38.59
Pinaceae	*Keteleeria davidiana*	NC_011930	117720	38.55
Cupressaceae	*Cryptomeria japonica*	NC_010548	131810	35.38
**Angiosperms:**				
Amborellaceae	*Amborella trichopoda*	NC_005086	162686	38.34
Ceratophyllaceae	*Ceratophyllum demersum*	NC_009962	156252	38.22
Nymphaeaceae	*Nymphaea alba*	NC_006050	159930	39.15
Nymphaeaceae	*Nuphar advena*	NC_008788	160866	39.12
Chloranthaceae	*Chloranthus spicatus*	NC_009598	157772	38.89
Schisandraceae	*Illicium oligandrum*	NC_009600	148553	39.05

Winteraceae	*Drimys granadensis*	NC_008456	160604	38.79
Calycanthaceae	*Calycanthus floridus var. glaucus*	NC_004993	153337	39.27
Magnoliaceae	*Liriodendron tulipifera*	NC_008326	159886	39.16
Piperaceae	*Piper cenocladum*	NC_008457	160624	38.31
Acoraceae	*Acorus calamus*	NC_007407	153821	38.60
Acoraceae	*Acorus americanus*	NC_010093	153819	38.59
Araceae	*Lemna minor*	NC_010109	165955	35.72
Orchidaceae	*Phalaenopsis aphrodite subsp. formosana*	NC_007499	148964	36.65
Dioscoreaceae	*Dioscorea elephantipes*	NC_009601	152609	37.15
Poaceae	*Saccharum hybrid cultivar SP-80-3280*	NC_005878	141182	38.44
Poaceae	*Saccharum officinarum*	NC_006084	141182	38.44
Poaceae	*Sorghum bicolor*	NC_008602	140754	38.49
Poaceae	*Zea mays*	NC_001666	140384	38.46
Poaceae	*Agrostis stolonifera*	NC_008591	136584	38.45
Poaceae	*Hordeum vulgare subsp. vulgare*	NC_008590	136462	38.32
Poaceae	*Triticum aestivum*	NC_002762	134545	38.31
Poaceae	*Lolium perenne*	NC_009950	135282	38.25
Poaceae	*Oryza nivara*	NC_005973	134494	39.01
Poaceae	*Oryza sativa Indica Group*	NC_008155	134496	39.00
Poaceae	*Oryza sativa Japonica Group*	NC_001320	134525	38.99
Poaceae	*Brachypodium distachyon*	NC_011032	135199	38.57
Poaceae	*Festuca arundinacea*	NC_011713	136040	38.41
Ranunculaceae	*Ranunculus macranthus*	NC_008796	155129	37.88
Berberidaceae	*Nandina domestica*	NC_008336	156599	38.32

Platanaceae	*Platanus occidentalis*	NC_008335	161791	38.03
Buxaceae	*Buxus microphylla*	NC_009599	159010	38.07
Amaranthaceae	*Spinacia oleracea*	NC_002202	150725	36.82
Polygonaceae	*Fagopyrum esculentum subsp. ancestrale*	NC_010776	159599	37.98
Vitaceae	*Vitis vinifera*	NC_007957	160928	37.40
Geraniaceae	*Pelargonium x hortorum*	NC_008454	217942	39.61
Myrtaceae	*Eucalyptus globulus subsp. globulus*	NC_008115	160286	36.86
Onagraceae	*Oenothera elata subsp. Hookeri*	NC_002693	165728	39.07
Onagraceae	*Oenothera argillicola*	NC_010358	165055	39.13
Onagraceae	*Oenothera biennis*	NC_010361	164807	39.07
Onagraceae	*Oenothera glazioviana*	NC_010360	165225	39.01
Onagraceae	*Oenothera parviflora*	NC_010362	163365	39.06
Salicaceae	*Populus alba*	NC_008235	156505	36.74
Salicaceae	*Populus trichocarpa*	NC_009143	157033	36.68
Euphorbiaceae	*Manihot esculenta*	NC_010433	161453	35.87
Euphorbiaceae	*Jatropha curcas*	NC_012224	163856	35.36
Fabaceae	*Glycine max*	NC_007942	152218	35.37
Fabaceae	*Phaseolus vulgaris*	NC_009259	150285	35.44
Fabaceae	*Medicago truncatula*	NC_003119	124033	33.97
Fabaceae	*Lotus japonicus*	NC_002694	150519	36.03
Fabaceae	*Cicer arietinum*	NC_011163	125319	33.91
Fabaceae	*Trifolium subterraneum*	NC_011828	144763	34.40
Moraceae	*Morus indica*	NC_008359	158484	36.37
Cucurbitaceae	*Cucumis sativus*	NC_007144	155293	37.08

Brassicaceae	*Arabidopsis thaliana*	NC_000932	154478	36.29
Brassicaceae	*Arabis hirsuta*	NC_009268	153689	36.40
Brassicaceae	*Aethionema cordifolium*	NC_009265	154168	36.62
Brassicaceae	*Aethionema grandiflorum*	NC_009266	154243	36.81
Brassicaceae	*Barbarea verna*	NC_009269	154532	36.43
Brassicaceae	*Capsella bursa-pastoris*	NC_009270	154490	36.55
Brassicaceae	*Crucihimalaya wallichii*	NC_009271	155199	36.42
Brassicaceae	*Olimarabidopsis pumila*	NC_009267	154737	36.48
Brassicaceae	*Draba nemorosa*	NC_009272	153289	36.47
Brassicaceae	*Lepidium virginicum*	NC_009273	154743	36.47
Brassicaceae	*Lobularia maritima*	NC_009274	152659	36.51
Brassicaceae	*Nasturtium officinale*	NC_009275	155105	36.37
Caricaceae	*Carica papaya*	NC_010323	160100	36.89
Rutaceae	*Citrus sinensis*	NC_008334	160129	38.48
Malvaceae	*Gossypium barbadense*	NC_008641	160317	37.23
Malvaceae	*Gossypium hirsutum*	NC_007944	160301	37.24
Rubiaceae	*Coffea arabica*	NC_008535	155189	37.43
Orobanchaceae	*Epifagus virginiana*	NC_001568	70028	36.00
Oleaceae	*Jasminum nudiflorum*	NC_008407	165121	37.98
Solanaceae	*Atropa belladonna*	NC_004561	156687	37.56
Solanaceae	*Nicotiana sylvestris*	NC_007500	155941	37.85
Solanaceae	*Nicotiana tabacum*	NC_001879	155943	37.85
Solanaceae	*Nicotiana tomentosiformis*	NC_007602	155745	37.79
Solanaceae	*Solanum bulbocastanum*	NC_007943	155371	37.88

Solanaceae	*Solanum lycopersicum*	NC_007898	155461	37.86
Solanaceae	*Solanum tuberosum*	NC_008096	155298	37.88
Convolvulaceae	*Ipomoea purpurea*	NC_009808	162046	37.48
Convolvulaceae	*Cuscuta gronovii*	NC_009765	86744	37.72
Convolvulaceae	*Cuscuta reflexa*	NC_009766	121521	38.22
Convolvulaceae	*Cuscuta exaltata*	NC_009963	125373	38.12
Convolvulaceae	*Cuscuta obtusiflora*	NC_009949	85286	37.84
Apiaceae	*Daucus carota*	NC_008325	155911	37.66
Araliaceae	*Panax ginseng*	NC_006290	156318	38.11
Asteraceae	*Lactuca sativa*	NC_007578	152765	37.55
Asteraceae	*Helianthus annuus*	NC_007977	151104	37.62
Asteraceae	*Guizotia abyssinica*	NC_010601	151762	37.62
Campanulaceae	*Trachelium caeruleum*	NC_010442	162321	38.33

[a] Sequenced chloroplast genome data were downloaded from NCBI Organelle Genome Resources (http://www.ncbi.nlm.nih.gov/genomes/GenomesGroup.cgi?taxid=2759&opt=plastid), except *Alsophila spinulosa* and *Selaginella uncinata*. The cp genome of *A. spinulosa* was presented in this study and that of *S. uncinata* was from GOBASE (http://megasun.bch.umontreal.ca/ogmp/projects/other/cp_list.html) [1]; GC contents and Genome sizes were calculated using BioEdit [2].

References:

1. O'Brien EA, Zhang Y, Wang E, Marie V, Badejoko W, Lang BF, Burger G: **GOBASE: an organelle genome database**. *Nucleic Acids Res* 2008, **37**:D946-950.
2. Hall TA: **BioEdit: a user-friendly biological sequence alignment editor and analysis program for Windows 95/98/NT**. *Nucleic Acids Symp Ser* 1999, **41**:95-98.
